# Follow-Up of Hearing Impairment in Patients with Congenital CMV Infection

**DOI:** 10.3390/children13020230

**Published:** 2026-02-06

**Authors:** Ron Fisher, Miriam Geal Dor, Cahtia Adelman, Michal Kaufmann-Yehezkely, Sagit Stern Shavit

**Affiliations:** 1 Department of Otolaryngology-Head and Neck Surgery, Hadassah Medical Center, Faculty of Medicine, Hebrew University of Jerusalem, Jerusalem 9112102, Israel; dkau375@hadassah.org.il (M.K.-Y.);; 2 Speech & Hearing Center, Hadassah Medical Center, Hebrew University of Jerusalem, Jerusalem 9112102, Israel; gmiriam@hadassah.org.il (M.G.D.); cahtiaa@hadassah.org.il (C.A.); 3 Department of Communication Disorders, Jerusalem Multidisciplinary College, Jerusalem 9422408, Israel

**Keywords:** congenital CMV, follow-up, hearing monitoring, delayed-onset SNHL, progressive hearing loss

## Abstract

**Highlights:**

**What are the main findings?**

• Delayed-onset SNHL in children with congenital CMV frequently develops after an initial period of normal hearing and may present over a broad age range.
• While poorer-ear thresholds are comparable, better-ear hearing remains more preserved in delayed-onset SNHL compared with congenital SNHL.

**What are the implications of the main findings?**

• cCMV-related hearing loss follows heterogeneous and asymmetric trajectories, with dynamic changes and progression in a substantial proportion of children.
• Detection and characterization of SNHL in cCMV depend on prolonged follow-up, underscoring both its clinical value and practical challenges.

**Abstract:**

**Background/Objectives**: Congenital cytomegalovirus (cCMV) is a leading non-genetic cause of childhood sensorineural hearing loss (SNHL), characterized by heterogeneous and dynamic hearing outcomes. Hearing impairment may be present at birth or emerge later in childhood. This study aimed to characterize hearing trajectories and laterality patterns in children with cCMV, with emphasis on congenital versus delayed-onset SNHL. **Methods**: We conducted a retrospective study of children with confirmed cCMV who underwent longitudinal audiologic follow-up. Hearing loss was classified as congenital SNHL or delayed-onset SNHL. Better- and poorer-ear thresholds, bilateral involvement, longitudinal changes, and follow-up duration were analyzed. **Results**: Of 195 included children, 59 (30%) developed SNHL. Congenital SNHL was present in 34 children (17%), while delayed-onset SNHL developed in 25 of 161 children (16%) who were born with normal hearing. Of these delayed-onset cases, 20 (80%) were asymptomatic at birth, while 5 (20%) presented with non-audiological neonatal symptoms. Longitudinal observation of the delayed-onset subgroup revealed that 36 ears developed SNHL during follow-up, spanning infancy through later childhood, including one case identified in early adulthood. Better-ear thresholds were significantly better preserved in delayed-onset SNHL, while poorer-ear thresholds were comparable across groups. Children with SNHL had substantially longer follow-up duration (60 ± 44.5 months) compared with those with normal hearing (37 ± 24.4 months). **Conclusions**: Children with cCMV-related SNHL exhibit dynamic and asymmetric hearing trajectories with clinically relevant differences between congenital and delayed-onset SNHL. These findings underscore the necessity of a risk-stratified, long-term surveillance framework that ensures individualized long-term monitoring and promotes sustained adherence to follow-up.

## 1. Introduction

Cytomegalovirus (CMV), a member of the *Herpesviridae* family, is the most common cause of congenital infection worldwide. The incidence of congenital cytomegalovirus (cCMV) varies globally, ranging from 0.2% to 2.0% of live births, with an average of 0.64% [1]. In Israel, the prevalence is estimated at approximately 0.7% [2].

Although most infected newborns are asymptomatic, cCMV is a leading cause of childhood sensorineural hearing loss (SNHL), accounting for about a quarter of all cases of hearing loss by age 4 years [3]. The clinical spectrum of cCMV is broad and heterogeneous. While 10–15% of newborns are symptomatic at birth—with up to half of them developing SNHL—the vast majority (85–90%) appear asymptomatic. Crucially, approximately 10–15% of these initially asymptomatic infants, who pass their newborn hearing screening, will still develop delayed-onset SNHL later in childhood [1,4,5,6], with lower rates reported in some large registry-based cohorts [7]. This distinction between congenital and postnatal manifestations is critical, as it underscores a prolonged window of vulnerability for the auditory system.

The biological mechanisms underlying CMV-related auditory damage remain incompletely understood [8]. Current evidence suggests that delayed-onset symptoms may arise from the reactivation of latent virus within the spiral ganglion or scala media [9]. Additionally, chronic inflammatory processes in the stria vascularis, characterized by persistent cytokine release, and progressive neural degeneration secondary to subclinical early viral injury have been proposed as key drivers of late-onset SNHL [10,11]. Together, these processes contribute to the unpredictable timing and progression of hearing loss, thereby justifying prolonged longitudinal audiologic surveillance in children with cCMV.

The risk of SNHL is influenced by maternal, viral, and neonatal factors. Regarding maternal infection, both the nature and timing of infection during gestation are critical. Primary maternal infection confers a substantially higher vertical transmission rate (approx. 30–40%) than non-primary infection (1–2%) [6,12]. Importantly, while the rate of vertical transmission increases as gestation progresses, the risk of severe fetal sequelae, including SNHL, is highest during the first trimester (approximately 22.8%) and decreases significantly when transmission occurs in the second or third trimesters [13].

Following transmission, the neonate’s clinical status serves as a key prognostic indicator. Several neonatal factors are associated with an increased risk of SNHL, including symptomatic disease at birth—characterized by findings such as petechiae, thrombocytopenia, hepatosplenomegaly or elevated liver enzymes [14], as well as high neonatal viral load [15] and neuroimaging abnormalities such as ventriculomegaly and white matter abnormalities [7,16].

Because early identification and intervention are critical for speech, language, and neurodevelopment, frequent repeated auditory monitoring for children with cCMV is essential [10]. However, existing guidelines differ in both the frequency and duration of follow-up, with recommendations ranging from surveillance until age 3–6 years [17,18] to continued monitoring into adolescence or adulthood [6,19]. Evidence supporting these extended schedules remains limited, and the optimal duration and intensity of surveillance remain unknown.

To address these gaps, this study aims to characterize long-term hearing trajectories and patterns of onset and progression in children with cCMV, while also evaluating real-world adherence to prolonged audiologic follow-up—key factors for informing evidence-based surveillance strategies.

## 2. Materials and Methods

We conducted a retrospective cohort study of children with cCMV born between the years 2005–2019 who were followed at Hadassah Medical Center between 2005 and 2025. Inclusion criteria were: (1) confirmed diagnosis of cCMV; (2) at least 4 documented audiologic assessments; and (3) follow-up period exceeding 1 year. The requirement for at least four assessments was used to ensure reliable longitudinal data within a single institution and to minimize loss of follow-up information. Diagnosis of cCMV infection was established by detection of CMV-DNA using polymerase chain reaction (PCR) in neonatal urine samples obtained within the first three weeks of life. We excluded children with known genetic causes of hearing loss or alternative clear etiologies for SNHL (e.g., meningitis, ototoxic chemotherapy), craniofacial anomalies, chronic inflammatory middle-ear disease, or those with incomplete audiologic data.

Clinical data were extracted from electronic medical records, and included birth weight, gestational age, neonatal intensive care unit (NICU) admission, neurologic signs, neuroimaging abnormalities, jaundice or hepatitis, thrombocytopenia, petechiae, pulmonary or ophthalmologic findings, and administration of antiviral therapy. Antiviral treatment (valganciclovir) was administered by a pediatric infectious disease specialist in children with cCMV-related systemic symptoms and/or sensorineural hearing loss.

### 2.1. Hearing Assessment

Audiological evaluations included both objective and behavioral tests. Initial objective assessments consisted of Transient Evoked Otoacoustic Emissions (TEOAE) and Automated Auditory Brainstem Response (AABR) performed prior to hospital discharge using Otodynamics system (Echocheck and Otoport; Otodynamics Ltd., Hatfield, UK).

At our institution, infants with confirmed cCMV follow a structured surveillance protocol. This includes an initial diagnostic ABR evaluation in early infancy, followed by audiologic monitoring every 3 months up to 18 months of age, every 6 months up to 3 years, and annually thereafter until age 7.

Diagnostic ABR thresholds were obtained in young infants using a Biologic Navigator-Pro Evoked Potential System (Natus Medical Inc., Mundelein, IL, USA) or an Interacoustics Eclipse system (Interacoustics, Middelfart, Denmark). Threshold was obtained in response to alternating stimuli, presented at a rate of 21.1 stimuli per second for click, and 39 stimuli per second for tonal stimuli at 1 kHz and 4 kHz. The stimuli consisted of at least 1024 sweeps with filter settings of 30–3000 Hz for clicks and 30–1500 Hz for tone bursts, using insert earphones and bone conduction as needed.

Behavioral assessments included visual reinforcement audiometry (VRA) for speech and tonal stimuli between 7 and 24 months, using insert earphones and bone conduction as needed. From approximately 2–3 years of age, conditioned play audiometry (CPA) and speech reception thresholds (SRTs) were obtained with a clinical audiometer (Interacoustics AC40, Assens, Denmark). When behavioral responses were unreliable or insufficient, sedated ABR was performed. Tympanometry was routinely used to assess middle-ear status.

Hearing impairment was classified by type (sensorineural or conductive), laterality (unilateral or bilateral), and severity: mild (20–40 dB), moderate (41–70 dB), severe (71–90 dB), or profound (>91 dB). Based on hearing status at birth and during follow-up, children were categorized as having congenital SNHL, delayed-onset SNHL, or normal hearing. Longitudinal hearing status was categorized as stable or deteriorated. Conductive hearing loss (CHL) was documented during follow-up. Children with isolated CHL were excluded from SNHL outcome analyses and grouped with children without SNHL for descriptive analyses, as the study focused on cCMV-related sensorineural hearing loss.

### 2.2. Statistical Analysis

Categorical variables are presented as counts and percentages and were compared using the chi-square test or Fisher’s exact test as appropriate. Pure-tone and ABR thresholds were treated as non-normally distributed and compared using the Mann–Whitney U test for both within-group (better vs. poorer ear) and between-group (congenital vs. delayed-onset SNHL) comparisons. Hearing thresholds and laterality were compared between treated and untreated children within the SNHL subgroup using the Mann–Whitney U and Fisher’s exact tests. The timing of delayed-onset SNHL was evaluated by plotting the cumulative number of newly diagnosed ears as a function of age at first detection. Each affected ear was included once, at the age when SNHL was initially identified. This analysis was used to describe the temporal distribution of delayed-onset SNHL across the follow-up period. Odds ratios (ORs) with 95% confidence intervals (CIs) were calculated for selected comparisons. Two-sided *p*-values < 0.05 were considered statistically significant. All statistical analyses were performed with GraphPad Prism 10.6.1.

## 3. Results

A total of 800 infants with cCMV were identified in our database. Of these, 198 met the inclusion criteria of having ≥4 documented audiologic assessments over more than one year of follow-up. Three children were excluded due to meningitis, hereditary immune disorder, and ototoxic chemotherapy, leaving a final cohort of 195 children.

**Figure 1:**
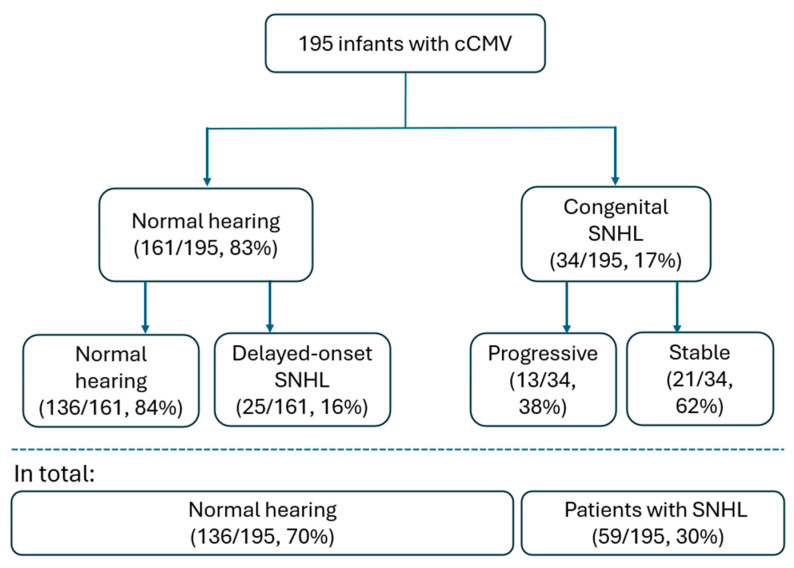
Flow diagram of hearing outcomes in children with congenital cytomegalovirus infection. Children were categorized according to hearing status at birth and during follow-up. Numbers indicate counts and percentages relative to the total cohort (n = 195).

Overall, 59 children (30%) developed SNHL. Among these, 34 (58%) had congenital SNHL and 25 (42%) developed delayed-onset SNHL. The remaining 136 children (70%) maintained normal hearing or only mild conductive hearing loss throughout follow-up. Among children with congenital SNHL, 21 (62%) remained stable, whereas 13 (38%) demonstrated progressive deterioration (Figure 1).

Average follow-up duration was 43.5 ± 33.5 months and extended beyond five years in 25% of children, between two and five years in 43%, and between one and two years in 32%.

Table 1 summarizes baseline characteristics across the three hearing-outcome groups (Congenital SNHL, Delayed-Onset SNHL, and Normal Hearing). Non-audiologic cCMV manifestations at birth (symptomatic cCMV infection) were documented in 52 of 195 (26.7%) infants and differed significantly between hearing-outcome groups (*p* = 0.00012). All children in the study underwent cranial ultrasound (US) after birth to screen for intracranial abnormalities. Of these, 43 children had abnormal neuroimaging findings; the most frequent were subependymal cysts (n = 18), followed by intracranial calcifications (n = 9), ventriculomegaly (n = 7), and microcephaly (n = 5). An additional 10 children exhibited other non-specific neuroimaging findings and developmental delay. Neurologic abnormalities were more common in the congenital SNHL group compared with the delayed-onset or the normal-hearing groups (47% vs. 20% and 16%, respectively; *p* = 0.00051). Jaundice and elevated liver enzymes showed a similar gradient, being substantially more frequent among infants with congenital SNHL (15% vs. 4% and 1.5%, respectively; *p* = 0.00234). Other birth findings were infrequent and did not differ significantly, although several trended higher in children with congenital SNHL.

Antiviral therapy was administered to 56 of 195 children (29%). Treatment was significantly more common in the congenital SNHL group (22/34, 65%) than in the delayed-onset SNHL group (9/25, 36%) or the normal hearing group (25/136, 18%; *p* = 0.037). However, statistical comparison revealed no significant differences in final hearing thresholds between treated and untreated children for either the poorer ear (*p* = 0.7015) or the better ear (*p* = 0.1628). Additionally, the proportion of bilateral versus unilateral SNHL was not significantly different between the groups (*p* = 0.0692).

Age at last audiologic assessment differed significantly by hearing outcome. Children with normal hearing had their last evaluation at a mean age of 37 ± 24.4 months, whereas children with SNHL (congenital or delayed-onset) were followed longer (mean 60 ± 44.5 months, mean difference 23 months, 95% CI 10.7–35.3; *p* = 0.0004).

Bilateral hearing involvement was present in 30 of 59 children (51%) with SNHL, 59% in the congenital SNHL group and 44% in the delayed-onset SNHL group (*p* = 0.192; OR 1.82, 95% CI 0.64–5.16).

**Figure 2:**
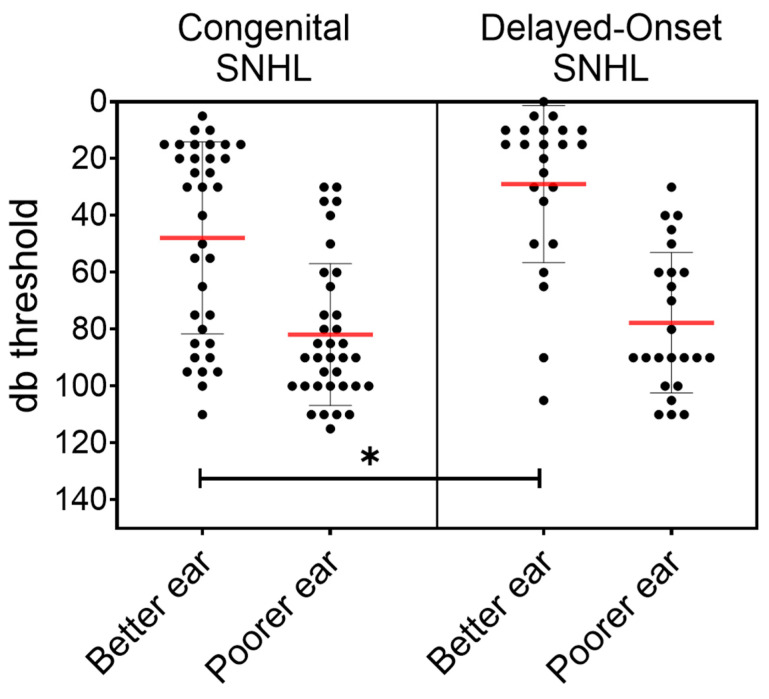
Better- and poorer-ear pure-tone thresholds in congenital and delayed-onset cCMV-related hearing loss. Individual ear thresholds are shown as scatter plots, grouped by clinical phenotype (**left**: congenital SNHL; **right**: delayed-onset SNHL) and by better versus poorer ear. Red horizontal lines represent group medians, and grey whiskers denote interquartile ranges. Within each group, poorer-ear thresholds were significantly higher than better-ear thresholds (Mann–Whitney, *p* < 0.05). Across groups, better-ear thresholds were significantly worse in the congenital SNHL group compared with the delayed-onset group (*p* = 0.0146), whereas poorer-ear thresholds did not differ between groups. * Indicates a statistically significant difference (*p* < 0.05).

Hearing thresholds were analyzed separately for the better and poorer ear, as only a minority of the children demonstrated symmetric loss. Figure 2 shows individual thresholds for congenital and delayed-onset SNHL. In both groups, the poorer-ear median threshold was 90 dB (IQR 70–100 dB). In contrast, better-ear thresholds differed significantly between groups, with median values of 30 dB (IQR 17.5–82.5 dB) in congenital SNHL and 15 dB (IQR 10–38.8 dB) in delayed-onset SNHL (*p* = 0.0146).

**Figure 3:**
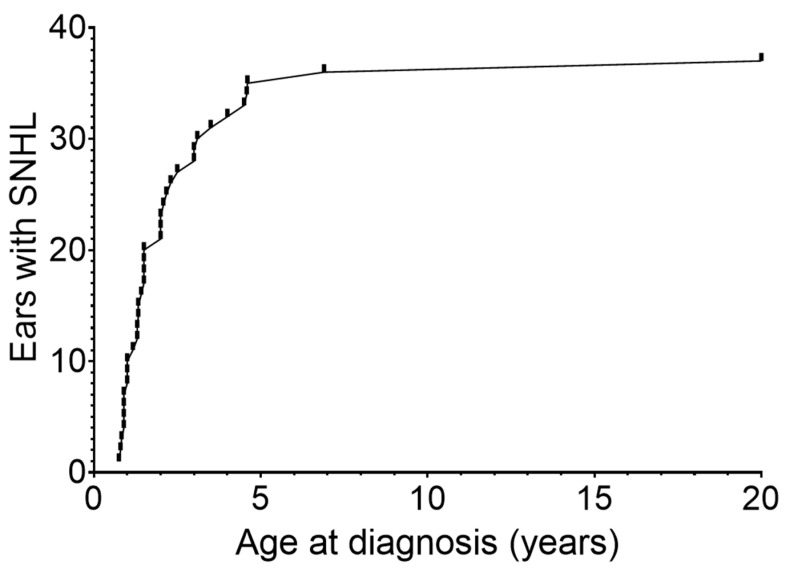
Cumulative number of ears diagnosed with delayed-onset SNHL by age at first detection. The curve depicts the cumulative count of ears in which delayed-onset SNHL was first identified during follow-up. Each increase represents a newly diagnosed ear at the corresponding age.

Timing of delayed-onset SNHL varied widely. Among the 25 children with delayed-onset SNHL, hearing loss developed in 37 of 50 ears (74%) during follow-up. Most diagnoses occurred early in life, with over half identified by two years of age. A further proportion was diagnosed between two and five years, and two ears (5%) were diagnosed after the age of five, including one case identified in adulthood (20 years). In these cases, a comprehensive diagnostic work-up was performed to rule-out alternative causes of SNHL. The cumulative distribution of age at first detection is shown in Figure 3.

## 4. Discussion

Congenital cytomegalovirus (cCMV) is a well-recognized cause of childhood sensorineural hearing loss (SNHL), yet the timing and progression of auditory impairment remain highly variable. The prevalence of SNHL in our cohort (30%) is consistent with previously reported rates ranging from 20% to 40% in both population-based and referral cohorts [5,20,21]. As described in prior studies, hearing loss was not confined to the neonatal period and could emerge or progress over time, including in children who initially passed newborn hearing screening.

We observed a relatively high proportion of symptomatic cases in our cohort (27%), exceeding the 10–15% typically reported in the literature [17,22]. This likely reflects the retrospective design and follow-up related selection bias, as children with early symptoms or clinical concerns are more likely to remain under longitudinal surveillance. Since April 2022, universal newborn cCMV screening has been implemented at our institution with structured audiologic follow-up [23], and future analyses of this prospective cohort are expected to provide a more representative estimate of symptom prevalence and long-term hearing trajectories.

Delayed-onset SNHL occurred in 16% of children with initially normal hearing, consistent with earlier reports describing delayed hearing loss in 10–15% of asymptomatic or mildly symptomatic infants with cCMV [5,21]. Although a recent large-scale European registry reported a lower incidence [7], this difference likely reflects variations in study design, follow-up duration, and surveillance intensity. In our cohort, most delayed-onset cases were identified within the first few years of life; however, a small number were diagnosed after the age at which routine follow-up is often discontinued. The frequent fluctuations and deteriorations observed in affected children, together with the occurrence of late-onset SNHL, underscore the prolonged and unpredictable risk window associated with cCMV-related hearing loss.

Delayed-onset and progressive SNHL in cCMV are increasingly understood as manifestations of ongoing pathological processes rather than static neonatal injury. Proposed mechanisms include reactivation of latent virus within the spiral ganglion or scala media, chronic or recurrent inflammatory activity within the stria vascularis, and progressive cochlear or neural degeneration following subclinical early viral injury [4,9,10,11]. These mechanisms provide a biological framework for the variable timing and progression observed in our cohort and support the rationale for longitudinal audiologic surveillance.

Analysis of ear-specific hearing thresholds demonstrated that poorer-ear thresholds reached comparable severity in both congenital and delayed-onset SNHL, whereas better-ear thresholds were more preserved in children with delayed-onset loss. This finding aligns with previous reports emphasizing asymmetric progression and earlier deterioration of the poorer-hearing ear in cCMV-associated SNHL [24]. Our data suggest that delayed-onset SNHL often follows a milder and more asymmetric course, while congenital SNHL more frequently involves bilateral and progressive disease, as reported in earlier cohorts [22,25].

Bilateral involvement was observed in 51% (30/59) of children with SNHL and in 59% of those with congenital SNHL, closely matching the 60–65% rates reported in systematic reviews of cCMV-associated congenital hearing loss [4]. Nearly half of children with congenital SNHL (47%) presented with severe-to-profound hearing loss at diagnosis, and 38% demonstrated progressive or fluctuating hearing thresholds over time, consistent with the 30–35% frequency of dynamic hearing patterns described in previous studies [5].

In our cohort, antiviral therapy was not significantly associated with final hearing outcomes within the SNHL subgroups. However, these findings should be interpreted with caution due to the non-randomized, retrospective nature of the study, where treatment was clinically indicated based on diverse symptoms and onset patterns.

A major clinical finding of this study relates to long-term follow-up adherence and its implications for surveillance strategies in children with cCMV. Nearly one-third of children discontinued audiologic follow-up within the first two years of life, most commonly those without early hearing impairment who nonetheless remain at risk for delayed-onset SNHL. Similar challenges have been reported in other cohorts and reflect real-world barriers to prolonged monitoring, including logistical, socioeconomic, and family-related factors [26,27,28].

At our institution, structured audiologic surveillance is conducted up to seven years of age; however, our findings demonstrate that hearing loss onset spans a broad age range, including cases identified beyond the period typically covered by routine follow-up programs. These observations highlight the need to balance the clinical benefit of early detection against the economic and practical burden of long-term surveillance. Rather than a rigid one-size-fits-all protocol, follow-up may benefit from a risk-stratified, family-centered approach that considers factors such as the trimester of maternal infection, primary versus non-primary infection status, neonatal symptom status, initial hearing thresholds, and the presence of language, motor, or vestibular deficits [5,16,26]. At the same time, our data demonstrate that delayed-onset SNHL can also occur in children initially considered low risk, underscoring the potential for missed diagnoses if surveillance is prematurely reduced. Long-term observational data, such as those presented here, together with future prospective studies and expert consensus, are therefore essential to define evidence-based, sustainable monitoring frameworks.

Vestibular findings were beyond the scope of the data available in this retrospective cohort, as standardized vestibular testing was not routinely performed. Nevertheless, vestibular dysfunction is increasingly recognized as a common and clinically significant manifestation of cCMV, with important implications for balance, motor development, and overall neurodevelopment, and it may occur independently of hearing status. Recent studies have highlighted the high prevalence of vestibular impairment and the risk of bilateral vestibulopathy in affected children [29,30]. Future prospective studies and follow-up protocols in children with cCMV should therefore incorporate systematic vestibular assessment alongside audiologic monitoring to provide a more comprehensive evaluation of neurosensory outcomes assessment [31].

This study has several limitations. Its retrospective design and reliance on available medical records may limit generalizability. Only a subset of children diagnosed with cCMV continued audiologic follow-up at our center, introducing selection bias. Children who were more closely monitored or at higher perceived risk were more likely to meet the inclusion criteria, while hearing outcomes for those lost to follow-up or monitored elsewhere were unavailable. Furthermore, the shorter follow-up duration in the normal-hearing group remains a significant limitation, as it may mask the eventual development of late-onset SNHL in some of these children. In addition, incomplete data on maternal serostatus and other perinatal or environmental risk factors limited comprehensive risk stratification. Regarding clinical interventions, the non-randomized allocation of antiviral therapy and variations in treatment duration preclude definitive conclusions regarding its efficacy.

Another limitation relates to the sensitivity of audiologic assessment in early infancy. Children with normal ABR, otoacoustic emissions, and early behavioral thresholds may still have subtle or frequency-specific deficits that are difficult to detect at young ages, raising the possibility of misclassification. Routine sedated ABR for all children with cCMV is neither clinically feasible nor ethically justified, underscoring the inherent challenges of early auditory evaluation in this population.

## 5. Conclusions

Children with cCMV-related SNHL demonstrate a wide range of hearing trajectories. Hearing loss may present early or late, progress asymmetrically, and evolve differently between ears. The better-ear advantage observed in delayed-onset SNHL, together with the greater tendency toward bilateral and progressive involvement in congenital SNHL, highlights important distinctions between clinical subgroups and reinforces the need for ongoing clinical vigilance throughout childhood. Our findings underscore the necessity of a risk-stratified, long-term surveillance framework that ensures individualized patient monitoring and promotes sustained adherence to follow-up throughout childhood, as missed evaluations may delay recognition of progressive or new hearing loss.

Future prospective studies are needed to establish evidence-based criteria for the duration and intensity of audiologic monitoring across different clinical scenarios, and to clarify how antiviral therapy and early risk markers influence long-term auditory outcomes in children with cCMV.

## Figures and Tables

**Table 1:** Baseline demographic and clinical characteristics of infants with congenital CMV, stratified by hearing outcome. Values are presented as n (%). *p*-values reflect comparisons across the three hearing outcome groups using the chi-square test. For antiviral treatment, Fisher’s exact test was used to compare the congenital SNHL and delayed-onset SNHL groups only. Abbreviations—SNHL, sensorineural hearing loss; IUGR, intrauterine growth restriction. * Statistically significant (*p* < 0.05).

Characteristic	Total (n = 195)	Congenital SNHL (n = 34)	Delayed-Onset SNHL (n = 25)	Normal Hearing (n = 136)	*p*-Value
Female	84 (43%)	17 (50%)	9 (36%)	58 (43%)	0.553
Antiviral treatment	56 (29%)	22 (65%)	9 (36%)	25 (18%)	0.037 *
Non-audiological symptoms at birth (Symptomatic at birth)	52 (27%)	19 (56%)	5 (20%)	28 (21%)	0.00012 *
Neurological findings	43 (22%)	16 (47%)	5 (20%)	22 (16%)	0.00051 *
Dermatological findings	2 (1%)	1 (3%)	1 (4%)	0 (0%)	0.090
Ocular findings	4 (2%)	2 (6%)	0 (0%)	2 (1%)	0.198
Jaundice/elevated liver enzymes	8 (4%)	5 (15%)	1 (4%)	2 (1%)	0.00234 *
Hematological abnormalities	12 (6%)	5 (15%)	1 (4%)	6 (4%)	0.073
Respiratory findings	6 (3%)	0 (0%)	0 (0%)	6 (4%)	0.261
IUGR	11 (6%)	3 (9%)	1 (4%)	7 (5%)	0.658
Prematurity (<37 wk)	21 (11%)	3 (9%)	0 (0%)	18 (13%)	0.135

## Data Availability

The datasets generated and analyzed during the current study are available from the corresponding author upon reasonable request due to ethical and privacy restrictions.

## Abbreviations

The following abbreviations are used in this manuscript:

ABR	Auditory Brainstem Response
AABR	Automated Auditory Brainstem Response
cCMV	Congenital Cytomegalovirus
CI	Confidence Interval
CPA	Conditioned Play Audiometry
HL	Hearing Loss
HNS	Head and Neck Surgery
IUGR	Intrauterine Growth Restriction
NICU	Neonatal Intensive Care Unit
OR	Odds Ratio
SNHL	Sensorineural Hearing Loss
SRT	Speech Reception Threshold
TEOAEs	Transient-Evoked Otoacoustic Emissions
VRA	Visual Reinforcement Audiometry
